# Molecular Patterns of Resistance Among *Helicobacter pylori* Strains in South-Western Poland

**DOI:** 10.3389/fmicb.2018.03154

**Published:** 2018-12-18

**Authors:** Aldona Bińkowska, Monika Maria Biernat, Łukasz Łaczmański, Grażyna Gościniak

**Affiliations:** ^1^2^nd^ Military Field Hospital of the Polish Armed Forces, Wrocław, Poland; ^2^Department of Hematology, Blood Neoplasms, and Bone Marrow Transplantation, Wrocław Medical University, Wrocław, Poland; ^3^Hirszfeld Institute of Immunology and Experimental Therapy, Polish Academy of Sciences, Wrocław, Poland; ^4^Department of Microbiology, Wrocław Medical University, Wrocław, Poland

**Keywords:** *H. pylori*, resistance, antibiotics, mutation, clarithromycin, levofloxacin

## Abstract

Treatment failure of *Helicobacter pylori* infection is caused mainly by progressive antibiotic resistance among *H. pylori* strains. In Poland, the prevalence of *H. pylori* strains resistant to metronidazole is higher than in other developed countries, reaching almost 50%, and resistance to clarithromycin is as high as 30% and is still increasing, contributing to the failure of first-line therapy in approximately 70% of patients. Moreover, the introduction of levofloxacin to eradication therapy of *H. pylori* infection quickly led to the emergence of resistant strains. Therefore, a necessary approach in microbiological diagnostics of *H. pylori* infection should be determination of susceptibility of *H. pylori* strains before the eradication treatment.

**Aim:** In this study was to evaluate the molecular mechanisms of resistance among 170 *H. pylori* strains to clarithromycin, involving mutations in the 23S rRNA gene (A2143G, A2142G, A2143G) and to levofloxacin, involving mutations of *gyrA* and *gyrB*. Analysis was performed by using polymerase chain reaction and classical sequencing of DNA fragments.

**Results:** Among examined strains, 26% were fully sensitive and 74% were resistant to at least one of the tested antibiotics. The overall resistance rate to metronidazole was as high as 56%, whereas to clarithromycin 46%, respectively. Resistance to LEV occurred among 6% of strains. All tested strains were susceptible to AMC and TET. The A2143G point mutation was found in 72% of clarithromycin-resistant strains. The most common mutation, present in 40% of *H. pylori* strains resistant to levofloxacin, was a change at position 91 of *gyrA*.

**Conclusion:** The increasing number of point mutations in the 23S rRNA gene leads to an increase in the rates of antimicrobial resistance. Presence of the GCG allele at position 122 of the *gyrA* gene may cause an eightfold increase in risk of development of resistance to levofloxacin.

## Introduction

*Helicobacter pylori* infection occurs most often in childhood, and the effects of infection can be seen usually in adulthood. *H. pylori* infection can lead to chronic gastritis, gastric and duodenal ulcers, gastric cancer, and MALT lymphoma. Standard therapy with a proton pump inhibitor and antibiotics such as clarithromycin and amoxicillin or metronidazole cures only about 70% of patients (Hunt et al., 2011; Mégraud, 2017). Although resistance to clarithromycin is considered the most important cause of ineffective *H. pylori* eradication, the causes of failure also include non-compliance with medical recommendations, high acidity in the stomach, a high load of bacteria and heterogeneity of *H. pylori* strains (Malfertheiner et al., 2012). The latest recommendations make treatment depend on the degree of resistance to clarithromycin. In regions with low resistance rates to clarithromycin (less than 20%), standard therapy, which contains clarithromycin, is recommended. In regions with high resistance to clarithromycin (>20%), first-line therapy with bismuth salts is recommended and, if it is not available, sequential therapy (PPI, amoxicillin for 5 days and PPI, clarithromycin, metronidazole, or tinidazole for the next 5 days) (Malfertheiner et al., 2012; Bartnik et al., 2014).

Resistance to clarithromycin arises as a result of point mutations in the domain V of the 23S rRNA gene of the 50S ribosomal bacterial subunit that leads to a change in the conformation of the target site. The change is so important that clarithromycin does not recognize the target site of action, and consequently is not able to stop the synthesis of proteins in the bacterial cell (Mégraud, 2004). Three mutations seem to be the most important: A2143G, A2142G, and A2142C. Transmission of resistance to clarithromycin takes place by horizontal gene transfer, in particular by acquisition of genes carried on mobile genetic elements from resistant to sensitive bacteria even within a strain of the same genotype (Wu et al., 2012). In addition, the exchange of genetic material can occur between different strains in mixed *H. pylori* infections (Taylor et al., 1997).

Until recently, rates of resistance of *H. pylori* strains to levofloxacin have been very low in Poland (Karczewska et al., 2014). However, the introduction of this drug into the treatment of *H. pylori* infections quite quickly resulted in the emergence of resistant strains. It seems that resistance to levofloxacin results from mutations in the quinolone resistance determining region (QRDR), where the genes *gyrA* and *gyrB*, encoding subunits of bacterial gyrase, are located. The point mutations which occur in the GyrA subunit are located in the following codons: 86, 87, 88, and 91 (Tankovic et al., 2003; Mégraud, 2004; Seck et al., 2013; Hanafi et al., 2016). The changes in the GyrB subunit, mainly at position 463, 438, 484 also should be taken into consideration (Miyachi et al., 2006; Hanafi et al., 2016). Unfortunately, there are *H. pylori* strains that despite the phenotypic resistance to levofloxacin, do not show any mutation in the QRDR region, neither the *gyrA* nor *gyrB* gene. This may indicate another mechanism of this resistance. Mutations in the *gyrA* and/or *gyrB* genes are present in approximately 83% of levofloxacin-resistant strains. It was suspected that mutations in the genes coding for topoisomerase IV parC or parE could be the cause of resistance, similar to *Neisseria gonorrhoeae* or mutations in the efflux system. However, so far no changes have been identified in these places that would be related to resistance to fluoroquinolones (Miyachi et al., 2006; Rimbara et al., 2012; Wang et al., 2012).

The resistance profile of the strains seems to be important in choosing an antibiotic for *H. pylori* eradication, especially after failure of the first line treatment (Taylor et al., 1997). The aim of this study was to identify point mutations in selected DNA fragments responsible for resistance of *H. pylori* strains using a sequencing technique.

## Materials and Methods

The research was carried out on 170 *H. pylori* strains, constituting part of the strain collection of the Department of Microbiology of Wrocław Medical University. The strains were isolated in the period 2008–2016 from children diagnosed and treated in the Second Chair and Department of Pediatrics, Gastroenterology and Nutrition, Wrocław Medical University, and from adults diagnosed in the Department and Clinic of Gastroenterology and Hepatology, and the Chair and Clinic of Gastrointestinal and General Surgery, Wrocław Medical University. The study was carried out in accordance with the World Medical Association’s Declaration of Helsinki. The protocol was approved by the Ethics Committee of Wrocław Medical University, Approval No. 154/2011. All subjects older than 16 years of age and parents of children under the age of 16, gave written informed consent to participate in the study. The strains were isolated from biopsies of the gastric mucosa taken during routine endoscopic examination of the upper gastrointestinal tract, which was performed due to clinical indications. The patients suffered from the following gastrointestinal complaints: dyspeptic symptoms, abdominal pain, nausea, and vomiting. All strains were isolated from previously untreated patients with primary *H. pylori* infection. After establishing diagnosis, patients were treated with standard regimen, consisting of proton pump inhibitor and two antibiotics: amoxicillin and clarithromycin or amoxicillin and metronidazole for 7 days. In case of detected resistance to clarithromycin and metronidazole, other regimens were used, including quadruple therapy with bismuth salts, and tetracycline or regimens based on levofloxacin in adults patients. The choice of antibiotic was dependent on the result of the susceptibility test (Koletzko et al., 2011; Bartnik et al., 2014; Iwańczak et al., 2016). The strains isolated from patients with recurrent *H. pylori* infection were not tested.

### Culture of Strains

Bacteriological examination of gastric mucosa samples was carried out on the basis of direct Gram staining, bacterial culture, and biochemical differentiation (urease, oxidase, and catalase test). Initially, the collected *H. pylori* strains, stored at -70°C in tryptic soy broth (TSB medium, Becton Dickinson, Germany) with the addition of 15% glycerol, were revived. After thawing, the strains were cultured on growth media by a method described elsewhere (Biernat et al., 2014). The strain *H. pylori* J99 from the collection of the Department of Microbiology of Wrocław Medical University was used as a reference strain.

### Susceptibility Testing by E-Test

The microorganisms obtained during the 72-h cultivation were then suspended in a brain heart infusion broth (BHI, Becton Dickinson, Germany). Cell concentration was determined using a densitometer (BioMeriux). Bacterial suspensions with a density of three according to the McFarland scale, i.e., 10^8^ cells (CFU)/1 ml were used for susceptibility testing. In subsequent steps, the susceptibility of the strains to the antibiotics amoxicillin (AMC), clarithromycin (CH), metronidazole (MTZ), tetracycline (TET), and levofloxacin (LEV) was determined by the E-test method using E-test strips (BioMerieux, Poland) as described elsewhere (Biernat et al., 2014). The MIC value of 32 μg/ml was considered as a cut-off point between low and high levels of antibiotic resistance (Versalovic et al., 1997).

### DNA Sequencing Genes From *H. pylori* Strains

From *H. pylori* strains obtained after 72 h of incubation, DNA was isolated by the column method using a Genomic Mini KIT (A&A Biotechnology, Poland). Then, polymerase chain reaction (PCR) was performed for amplification regions, including following genes: *23S rRNA* (DNA fragment corresponding to position 1881–2150), *gyrA* (DNA fragment corresponding to position 121–588), *gyrB* (DNA fragment corresponding to position 1153–1560). Based on the sequence of reference strain J99 available on the GenBank website, the following primers were designed to detect point mutations: 23S rRNA forward primer (5′-AAT TGA AGC CCG AGT AAA CG-3′), 23S rRNA reverse primer (5′-ATG GCT CCA TAA GAG CCA AA-3′), gyrA forward primer (5′-AGC TTA TTC CAT GAG CGT GA-3′), gyrA reverse primer (5′-TCA GGC CCT TTG ACA AAT TC-3′), gyrB forward primer (5′-CCC TAA CGA AGC CAA AAT CA-3′), gyrB reverse primer (5′-GGG CGC AAA TAA CGA TAG AA-3′). The oligonucleotides complementary to the individual *H. pylori* genomic DNA fragments (Generi Biotech, Czechia) and the TaKaRa Taq Hot Start Version PCR kit (TaKaRa, Co., Japan) were used. The product of the 23S rRNA gene was amplified under the following conditions: 5 min at 94°C for initial denaturation followed by 35 cycles of 30 s at 94°C, 30 s at 57°C, and 30 s at 72°C with a final round of 5 min at 72°C. The size of the product obtained was 270 bp. Conditions of PCR reaction for *gyrA* and *gyrB* genes were as follows: 10 min at 95°C for initial denaturation followed by 35 cycles of 30 s at 95°C, 45 s at 58°C, and 30 s at 72°C with a final round of 5 min at 72°C. The size of the product obtained for *gyrA* and *gyrB* was 467 and 407 pz, respectively. The PCR products were then purified from the remaining free nucleotides and primers of the reaction with a mixture of exonuclease enzymes (Exo I) and alkaline phosphatase (SAP) in a 2:1 ratio. The sequencing reaction was performed using the BigDye Terminator v3.1 Cycle Sequencing Kit (Applied Biosystems, United States) in the presence of primers complementary to the sequences of the tested genes using the standard conditions described by the manufacturer. Sequence analyses were performed using the ABI 3500 sequencer. The results were visualized using the FinchTV program. Each sequence was compared to the sequences of *H. pylori* J99 and *H. pylori* 26695 reference strains, available on the GenBank website.

### Statistical Analyses

The results of the study were analyzed statistically by Pearson′s χ^2^ test, maximum likelihood χ^2^ test, Yates test, logistic regression method (multivariate analysis), and Spearman′s rank correlation test. In addition, the Mann–Whitney *U*-test was used to evaluate the effect of the number of mutations on resistance, in which all data are compared to each other. A *p*-value ≤ 0.05 was considered statistically significant.

## Results

In this study, 170 *H. pylori* strains, isolated from children (*n* = 123) and adults (*n* = 47) with various diseases of the gastrointestinal tract, such as chronic gastritis, and peptic or duodenal ulcer, were tested. Among all strains, 26% were susceptible and 74% were resistant to at least one of the tested antibiotics (Table 1). Resistance to MTZ occurred among 56% of strains, 24% of strains were resistant only to MTZ, whereas 32% of them were multi-resistant. Among all strains, 46% were resistant to CH, whereas 17% were resistant only to CH, and multi-resistant strains accounted for 29%. Resistance to LEV occurred among 6% of strains, whereas 1% were resistant to LEV only, and multiresistant strains accounted for 5%. All tested strains were susceptible to AMC and TET.

**Table 1:** Resistance rates of examined *H. pylori* strains.

Antibiotics	Total		Children		Adults	
	*n*	*%*	*n*	*%*	*n*	*%*
MTZ *(total)*	96	56	59	48	37	79
CH *(total)*	79	46	64	52	15	32
LEV *(total)*	10	6	2	2	8	17
MTZ	41	24	24	20	17	36
CH	29	17	29	24	0	0
LEV	1	1	0	0	1	2
MTZ+CH	46	27	33	27	13	28
MTZ+CH+LEV	4	2	2	2	2	4
MTZ+LEV	5	3	0	0	5	11
AMC	0	0	0	0	0	0
TET	0	0	0	0	0	0
Susceptibility strains	44	26	35	28	9	19
Total	170	100	123	100	47	100


*n, number of resistant strains; %, percent of resistant strains; MTZ, metronidazole; CH, clarithromycin; LEV, levofloxacin; TET, tetracycline; AMC, amoxicilin.*

In the next step, 106 of 170 *H. pylori* strains, with a known clarithromycin susceptibility profile, were selected for further studies. We chose only those strains which were passaged for only two or three times on culture media to reduce the risk of high genetic variability of analyzed regions (Odenbreit et al., 2009; Hanafi et al., 2016).

**FIGURE 1:**
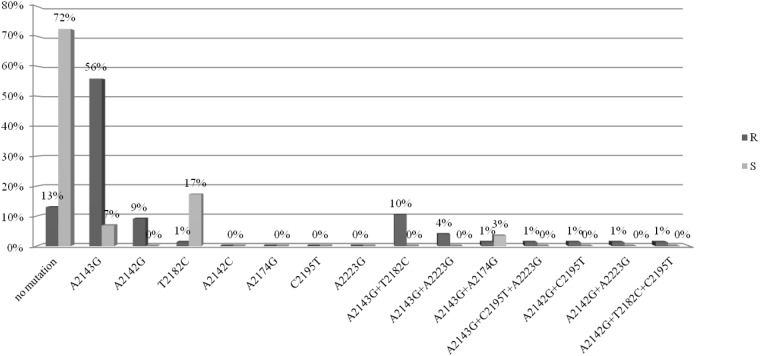
Distribution of point mutations in 23S rRNA gene in *H. pylori* strains susceptible and resistance to clarithromycin.

They were isolated from 91 children and 15 adults. The analysis of mutations in the *23S rRNA* gene was performed for 77/106 strains resistant to clarithromycin (study group) and for 29/106 susceptible strains (control group). The point mutations in the *23S rRNA* gene in *H. pylori* strains resistant to clarithromycin were tested (Figure 1). Among these strains, 72% (56/77) had a mutation at position A2143G, of which 56% (43/77) had a single mutation, while the other strains had at least two mutations. In 10% (8/77) of strains, mutations at positions A2143G and T2182C were detected. Among strains resistant to CH, 12% (10/77) had a mutation at position A2142G, of which 9% (7/77) had a single mutation, while the others had at least two mutations. No mutations were found in 13% of strains from the study group.

The point mutations in the *23S rRNA* gene of *H. pylori* strains sensitive to clarithromycin are shown in Figure 1. Among strains sensitive to clarithromycin, 10% (3/29) had a mutation at position A2143G, of which 7% (2/29) had a single mutation, and 3% (1/29) possessed mutations A2143G and A2174G. In addition, mutations at position T2182C were detected in 17% (5/29) of strains. The remaining 72% (21/29) of strains from the control group did not have any mutations in the *23S rRNA* gene. Fluorograms showing mutations detected in the *23s rRNA* gene by the sequencing method are presented in Supplementary Material.

**FIGURE 2:**
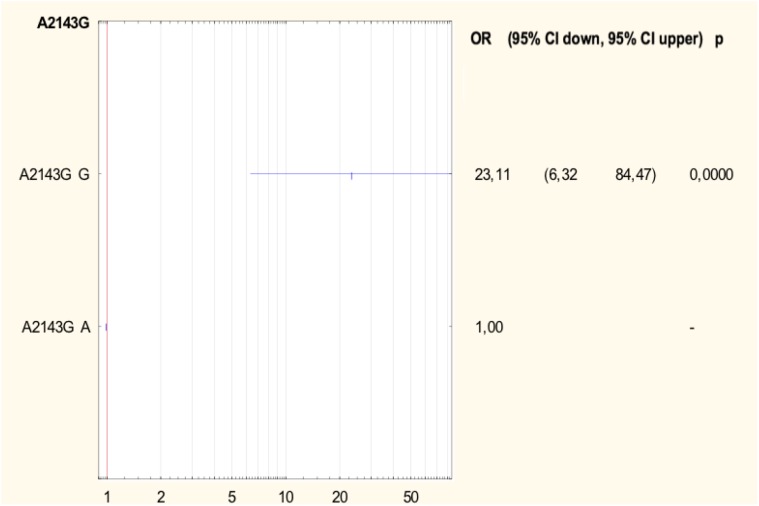
Forest plot describing the odds ratio of resistance to clarithromycin for mutation A2143G. OR, odds ratio; p, *p*-value.

Half of all strains with the A2142G mutation were characterized by a MIC value ≤ 32, while other strains had a MIC value > 32 (Table 2) (Versalovic et al., 1997). However, 57.1% (4/7) of strains with a single A2142G mutation had MIC ≤ 32 and 42.9% (3/7) of them had MIC > 32. Strains with the A2142G + C2195T mutations were characterized by MIC ≤ 32, whereas the mutations A2142G + A2223G and A2142G + T2182C + C2195T had MIC > 32. The MIC ≤ 32 value was found in 83.9% (47/56) of all *H. pylori* strains resistant to clarithromycin with the A2143G mutation, and in 86% (37/43) of them with a single A2143G mutation. The value of MIC > 32 occurred in 16.1% (9/56) of all strains with the mutation A2143G, 14% (6/43) of strains with the single mutation A2143G and in 37.5% (3/8) of strains with two mutations: A2143G + T2182C. Strains resistant to clarithromycin with the T2182C mutation had MIC ≤ 32. In contrast, 80% (8/10) of resistant strains, not having mutations in the studied fragment, were characterized by MIC ≤ 32, and the remaining 20% (2/10) of them were characterized with MIC > 32. There was no statistically significant difference between the groups (χ^2^ = 19.910, *p* = 0.0688). Using statistical analysis it was shown that the strains with higher MIC values more often possessed a mutation at position A2143G (*p* = 0.00025 and *p* < 0.001). Below OR (odds ratio) = 23.11, the risk of resistance was 23x higher among strains with the G mutation of the A2143G mutation than wild-type strains (Figure 2). Together with the increase in the number of mutations, the percentage of susceptible strains was significantly lower (Pearson’s χ^2^ 36.24573, *p* = 0.00000 and maximum likelihood χ^2^ test 35.34400, *p* = 0.00000). The occurrence of at least one (any) mutation increases the risk of resistance (Pearson’s χ^2^ 35.95308, *p* = 0.00000, maximum likelihood χ^2^ test 34.49099, *p* = 0.00000, Yates’ test 33.13852, *p* = 0.00000). The presence of A2143G mutation increased the risk of resistance about 17-fold (OR = 17.587). There was a significant relationship between the number of mutations and the degree of resistance (Pearson’s χ^2^ 98.28355, *p* = 0.00611 and maximum likelihood χ^2^ test 96.03649, *p* = 0.00928). In addition, a statistically significant relationship was demonstrated between the locus of mutation and resistance, e.g., the mutation at position 2143 was crucial for resistance to clarithromycin (Pearson’s χ^2^ 55.58826, *p* = 0.00000 and maximum likelihood χ^2^ test 60.87119, *p* = 0.00000).

**Table 2:** Correlation between MIC value (mg/L) and type of mutation in *23S rRNA* gene among *H. pylori* strains resistant to clarithromycin.

Mutation	MIC (mg/L)			
	≤32		>32	
	*n*	*%*	*n*	*%*
A2142G all	5	50,0	5	50,0
A2142G single	4	57,1	3	42,9
A2142G+C2195T	1	100,0	0	0,0
A2142G+A2223G	0	0,0	1	100,0
A2142G+T2182C+C2195T	0	0,0	1	100,0
A2143G all	47	83,9	9	16,1
A2143G single	37	86,0	6	14,0
A2143G+A2174G	1	100,0	0	0,0
A2143G+T2182C	5	62,5	3	37,5
A2143G+A2223G	3	100,0	0	0,0
A2143G+C2195T+A2223G	1	100,0	0	0,0
T2182C	1	100,0	0	0,0
No mutation	8	80,0	2	20,0


**FIGURE 3:**
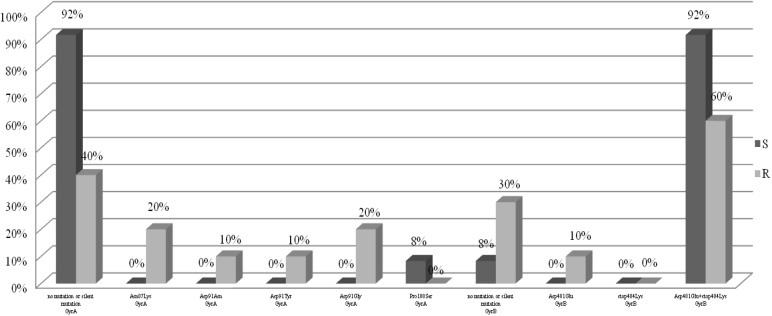
Distribution of sense mutations in *gyrA* and *gyrB* gene *H. pylori* strains susceptible and resistant to levofloxacin.

For the analysis of the mutations in the *gyrA* and *gyrB* genes, 22 *H. pylori* strains were selected, including 10 strains resistant to levofloxacin as the test group and 12 sensitive strains as a control group. Figure 3 shows the point mutations in the *gyrA* and *gyrB* gene in *H. pylori* strains susceptible and resistant to levofloxacin. Among resistant strains, 20% (2/10) had the Asn87Lys mutation, another 20% (2/10) had the Asp91Gly mutation, 10% (1/10) had the Asp91Asn mutation and 10% (1/10) had the Asp91Tyr mutation. 40% (4/10) of tested strains did not have any sense mutation or had silent mutations. Among susceptible strains, 8% (1/12) had Pro188Ser mutations and the remaining 92% (11/12) did not have any sense mutation or silent mutations. Among resistant strains 60% (6/10) possessed mutations Asp481Glu + Stop484Lys, and another 10% (1/10) had the mutation Asp481Glu. 30% (3/10) of resistant strains did not have any sense mutation or had silent mutations. Among sensitive strain, 92% (11/12) possessed mutations Asp481Glu + Stop484Lys, and the remaining 8% (1/12) did not have any sense mutation or had silent mutations.

**FIGURE 4:**
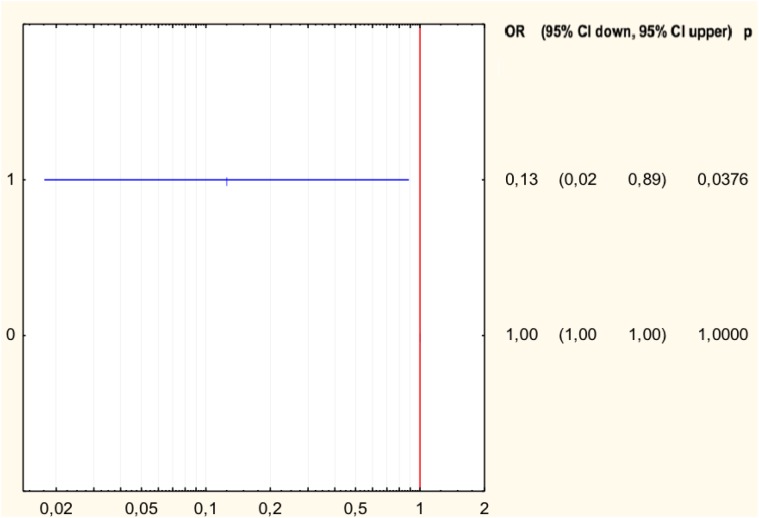
Forest plot describing the odds ratio of resistance to levofloxacin for mutation Ile191Met. OR, odds ratio; p, *p*-value.

The analysis of the relationship between the MIC values and the type of mutation in *H. pylori* strains resistant to levofloxacin was performed (Table 3). Half of all strains with the Asp91Gly mutation were characterized by MIC = 32, while the remaining strains showed MIC = 4. However, the Asp91Tyr mutant strain was characterized by a high MIC = 32 and the strain with the Asp91Asn mutation had MIC = 4. All Asn87Lys mutant strains had MIC = 32. The mutant strains were characterized by MIC from low (3) to high (32). After applying Spearman′s correlation test, it was found that the correlation between the number of mutations and resistance to levofloxacin was not significant (*p* = 0.064350). The presence of isoleucine at position 191 of the *gyrA* gene significantly reduced the risk of resistance to fluoroquinolones by 87% (*p* = 0.0376) (Figure 4). The presence of the GCG allele at position 122 of the gyrA gene significantly increased the risk of resistance to levofloxacin eightfold (*p* = 0.0376). There was a significant relationship between the number of mutations and resistance to levofloxacin. We found that strains resistant to this antibiotic had a higher mean number of mutations than did sensitive strains (Mann–Whitney test, *p* = 0.0358).

**Table 3:** Correlation between MIC value (mg/L) and type of mutation in *gyrA* gene among *H. pylori* strains resistant to levofloxacin.

Mutation	MIC	*n*	*%*
Asn87Lys	32	2	100
Asp91Asn	3	1	100
Asp91Tyr	32	1	100
Asp91Gly	4	1	50
	32	1	50
No mutation	3	2	50
	8	1	25
	32	1	25


## Discussion

Resistance of *H. pylori* strains poses a serious therapeutic problem worldwide and contributes to the failure of eradication of *H. pylori* infection in approximately 70% of patients (Mégraud, 2004; Iwańczak et al., 2014). The World Health Organization listed *H. pylori* strains resistant to clarithromycin in the group of the most dangerous alert pathogens (Tacconelli et al., 2018).

In this study, 170 *H. pylori* strains were tested; 26% of them were susceptible, and 74% were resistant to at least one of the tested antibiotics. Multiresistant strains accounted for as many as 32%, and included those resistant to two (metronidazole and clarithromycin or metronidazole and levofloxacin) or three (metronidazole, clarithromycin, and levofloxacin) antibiotics. In Poland, as well as in Eastern and Central European countries, in Nigeria, Southeast Asia, China and Argentina, resistance to clarithromycin is high and ranges from about 15 to even 30% (Mégraud, 2004; Hunt et al., 2011; Iwańczak et al., 2014). Such high resistance of strains to clarithromycin in Poland results mainly from excessive consumption of antibiotics, especially from the group of macrolides, and their widespread use in respiratory tract infections. In our study, the most frequently occurring mutation in *H. pylori* strains resistant to clarithromycin was the A2143G mutation in the *23S rRNA* gene, which was present in 72% of resistant strains, which confirms previous observations of other authors from Poland (Raymond et al., 2007; Klesiewicz et al., 2014). We have demonstrated that the risk of resistance to this group of antibiotics is 23 times higher among strains that have the mutation A2143G. Many researchers consider it as the main cause of resistance to clarithromycin (Taylor et al., 1997). Raymond et al. analyzed *H. pylori* strains isolated from children in France which were resistant to clarithromycin and, in 90% of them, the A2143G mutation was identified (Raymond et al., 2007). According to Mégraud (2004), 61% of *H. pylori* strains resistant to clarithromycin possess the A2143G mutation. In addition, other mutations such as T2182C, A2223G, T2244C, A2302G, C2195T, and C1953T were also common in *H. pylori* strains (Phan et al., 2015a,b). In northern China, three other mutations, G2224A, C2245T and T2289C, were identified in *H. pylori* strains resistant to clarithromycin (Vaziri et al., 2013). In our study, other mutations in the *23S rRNA* gene of *H. pylori* strains resistant to clarithromycin (T2182C, A2223G, A2174G, and C2195T) were observed also. Most often, they occurred together with the A2143G mutation. In our study, we found that the probability of the presence of susceptible strains significantly decreases with the increase in the number of mutations in the *23S rRNA* gene (*p* < 0.00001), and the introduction of even one additional mutation increases the risk of resistance to clarithromycin (*p* < 0.000001) and affects the degree of resistance. There is no single position on the significance of the T2182C mutation in creating a mechanism of resistance to clarithromycin. However, in our study, as in those of other authors, the binding mutation was associated with a low degree of resistance to clarithromycin (De Francesco et al., 2006b; Ierardi et al., 2013; Vaziri et al., 2013). It is also surprising that in more than 10% of the tested strains sensitive to clarithromycin with a MIC value of 0.016 the A2143G mutation was identified also. The reason for this phenomenon may be related to the existence of another feature, which appears only when combined with the A2143G mutation leading to the resistance phenotype, but this observation requires further research. It should be noted that among *H. pylori* tested strains resistant to clarithromycin, there were also 12% of strains with the A2142G mutation. In Poland, the incidence of A2142G mutations in resistant strains was 42.9% (Klesiewicz et al., 2014). In Egypt a mutation at this position was observed more often, in 57.7% of strains resistant to clarithromycin (Ubhayawardana et al., 2015). In Vietnam, however, the A2142G and A2142C mutations were not found (Caliskan et al., 2015; Phan et al., 2015b). The occurrence of various mutations determining resistance to clarithromycin is not fully understood. It may result from the variability of strains in humans and their genetic variations (Caliskan et al., 2015; Phan et al., 2015b). The relationship between the MIC value and the type of mutation in the 23S rRNA gene was also examined. Versalovic proposed the MIC value of 32 as a cut-off point between low and high levels of antibiotic resistance (Versalovic et al., 1997). In the present study, a high MIC value was observed for half of all strains with the A2142G mutation and about 43% of strains with a single A2142G mutation. The MIC value was also influenced by the presence of other mutations, e.g., strains A2142G + A2223G and A2142G + T2182C + C2195T had a MIC value > 32. However, the vast majority of strains (83.9%) with the A2143G mutation had MIC ≤ 32, and still more were identified with the single A2143G mutation (86%). Other studies conducted in Poland also showed that strains with the A2142G mutation correlated with high resistance to clarithromycin, while those with the A2143G mutation were characterized by lower MIC values (Klesiewicz et al., 2014). Chen indicated that A2142G and A2142C mutations were associated with a high degree of resistance to macrolides, considering the cut-off point MIC = 64 (Chen et al., 2018). The A2143G mutation is not only associated to high MIC value, but also may increase the risk of therapy failure (De Francesco et al., 2006a). Although in some studies strains with the A2142G mutation show a higher degree of resistance to this drug, the cause of this phenomenon has not yet been identified (Vaziri et al., 2013). Strains resistant to clarithromycin with the T2182C mutation had MIC ≤ 32. In contrast, 80% of resistant strains, but not having mutations in the studied fragment, were characterized by MIC ≤ 32, and the remaining 20% had MIC > 32. In this study, in 13% of *H. pylori* strains resistant to clarithromycin, mutations in the 23S rRNA gene were not detected, which may suggest another mechanism of resistance. In addition to the 23S rRNA mutation, another mechanism of resistance to clarithromycin, associated with expression of the RND efflux pumps (resistance-nodulation-cell division), has been described, The efflux pump system is not the primary and main cause of resistance to clarithromycin (Chen et al., 2018), but a secondary mechanism found in multiresistant strains. It should also be noted that most of these strains were characterized by high or medium MIC, e.g., MIC 256 – 20% (2/10), MIC 32 – 10% (1/10), MIC 24 – 10% (1/10 ), MIC 8 – 20% (2/10), and MIC 4 – 40% (4/10) of the strains.

Currently, in many places in the world, including in Poland, an increase in the resistance of *H. pylori* to levofloxacin is observed. According to Karczewska, in 2006–2008, resistance to levofloxacin was as high as 5%, and by 2009–2011 it had increased to 16%. In studies from Lower Silesia, resistance to levofloxacin was 8% (Biernat et al., 2014; Karczewska et al., 2014). Similarly, in Spain and France, the number of resistant strains increased from 6 and 3% to 25 and 17% respectively over several years (Hunt et al., 2011). In our study, among all tested strains, 6% were resistant to levofloxacin, with 5% of strains being multiresistant. The relatively low frequency of resistance to levofloxacin among the strains studied may be related to the fact that the strains were predominantly isolated from children, a population in which fluoroquinolones are used very rarely.

The resistance of *H. pylori* to fluoroquinolones is most commonly associated with a mutation in the *gyrA* gene in codons 87 and 91 (Hanafi et al., 2016). Moore described the following mutations in the *gyrA* gene of levofloxacin-resistant *H. pylori* strains: Asn87Lys, Ala88Val, Asp91Gly, Asp91Asn, Asp91Tyr, and the Ala91Val and Ala97Val double substitution (Moore et al., 1995). In our study, among the fluoroquinolone-resistant *H. pylori* strains, 20% had mutations in codon 87, where the change of asparagine to lysine occurred. Most often, however, mutations in codon 91 were observed, which occurred in 40% of strains. In France, the mutation at position Asn87Lys in the gene *gyrA* occurred in 27% of *H. pylori* strains, and the Thr87Tyr mutation in 9%. The Asp91Asn mutation occurred in 36% of French strains and Asp91Tyr in 14% (more often than in this study). Interestingly, higher MIC values were found in strains with the Asn-87 mutation (Phan et al., 2015a,b). MIC was higher in strains that were resistant to clarithromycin and levofloxacin (Phan et al., 2015a). In our study, all strains with mutations at position 87 were characterized by high MICs, while strains with mutations at position 91 were more diverse. Depending on the type of mutation, there was another MIC, but there were too few of them to draw conclusions. In Vietnam, a new combination of mutations in the levofloxacin resistant strain – Asn87Ala, Ala88Asn, and Val65Ile was found (Phan et al., 2015a). In our study, 40% of *H. pylori* resistant to levofloxacin did not have any sense mutation or had silent mutations. Despite this, it turns out that the change of one nucleotide may affect the resistance of the strains. It was found that the presence of GCG alleles at position 122 of the *gyrA* gene increases the risk of resistance to levofloxacin eightfold (*p* = 0.0376). In French studies, only 1% of strains did not have any mutations (Garcia et al., 2012). However, there is a relationship between the number of mutations and resistance to levofloxacin. It was found that strains resistant to this antibiotic have a higher mean number of mutations than sensitive strains (*p* = 0.0358). In addition, the presence of isoleucine at position 191 of the *gyrA* gene statistically significantly reduced the risk of resistance to fluoroquinolones by 87% (*p* = 0.0376). Among the sensitive strains, 8% had Pro188Ser mutations in the *gyrA* gene, and the remaining 92% did not have any sense mutation or had silent mutations. Probably there is another mechanism, such as the efflux system, which is responsible for high resistance to levofloxacin (Cambau et al., 2009). In our study, resistant and sensitive strains to levofloxacin had mutations in the *gyrB* gene Asp481Glu + Stop484Lys, respectively 60 and 92%. The Asp481Glu mutation occurred only in 10% of resistant strains. Silent mutations or lack of sense mutations were characteristic for 30% of resistant strains and for 8% of sensitive strains. This means that probably, not the mutations in the *gyrB* gene, but rather random event has the greatest impact on the emergence of resistance to levofloxacin. Our research has some limitations. We analyzed only some selected mutations related to phenotypic resistance. We believe that further research studies are needed to explain multiple phenotypes related to resistance in clinical practice.

## Conclusion

Among the tested *H. pylori* strains isolated from patients with primary infections, up to 32% were simultaneously resistant to at least two antibiotics used as standard in eradication therapy. Thanks to the applied methods of sequencing it was possible to analyze not one but many genetic changes within the examined genes. Among *H. pylori* strains resistant to clarithromycin, mutations at positions A2143G, A2142G, and A2143G + T2182 were most frequently found. The risk of resistance to clarithromycin is significantly higher among *H. pylori* strains with the A2143G mutation. With the increase in the number of mutations, the percentage of *H. pylori* strains sensitive to clarithromycin significantly decreases. The presence of the GCG allele at position 122 of the *gyrA* gene increases the risk of resistance to levofloxacin, while the presence of isoleucine at position 191 of the *gyrA* gene reduces the risk of resistance to levofloxacin.

## Author Contributions

AB, MB, and GG conceived the study. AB and MB performed the culture and antibiotic testing. AB and ŁŁ conducted the PCR analysis and sequencing. All authors participated in the critical review of the manuscript.

## Conflict of Interest Statement

The authors declare that the research was conducted in the absence of any commercial or financial relationships that could be construed as a potential conflict of interest.

## References

[B1] Bartnik W. Celińska-Cedro D. Dzieniszewski J. Łaszewicz W. Mach T. Przytulski K. (2014). Guidelines from the polish society of gastroenterology for the diagnosis and treatment of *Helicobacter pylori* infection. *Gastroenterol. Prakt.* 2 33–41.

[B2] Biernat M. M. Poniewierka E. BŁaszczuk J. Czapla L. Kempiński R. Ksiądzyna D. (2014). Antimicrobial susceptibility of *Helicobacter pylori* isolates from Lower Silesia, Poland. *Arch. Med. Sci.* 10 505–509. 10.5114/aoms.2013.36917 25097581 PMC4107243

[B3] Caliskan R. Tokman H. B. Erzin Y. Saribas S. Yuksel P. Bolek B. K. (2015). Antimicrobial resistance of *Helicobacter pylori* strains to five antibiotics, including levofloxacin, in Northwestern Turkey. *Rev. Soc. Bras. Med. Trop.* 48 278–284. 10.1590/0037-8682-0027-2015 26108005

[B4] Cambau E. Allerheiligen V. Coulon C. Corbel C. Lascols C. Deforges L. (2009). Evaluation of a new test, genotype HelicoDR, for molecular detection of antibiotic resistance in *Helicobacter pylori*. *J. Clin. Microbiol.* 47 3600–3607. 10.1128/JCM.00744-09 19759218 PMC2772597

[B5] Chen J. Ye L. Jin L. Xu X. Xu P. Wang X. (2018). Application of next-generation sequencing to characterize novel mutations in clarithromycin-susceptible *Helicobacter pylori* strains with A2143G of 23S rRNA gene. *Ann. Clin. Microbiol. Antimicrob.* 17:10. 10.1186/s12941-018-0259-8 29562911 PMC5863438

[B6] De Francesco V. Margiotta M. Zullo A. Hassan C. Troiani L. Burattini O. (2006a). Clarithromycin-resistant genotypes and eradication of *Helicobacter pylori*. *Ann. Intern. Med.* 144 94–100. 16418408 10.7326/0003-4819-144-2-200601170-00006

[B7] De Francesco V. Margiotta M. Zullo A. Hassan C. Valle N. D. Burattini O. (2006b). Primary clarithromycin resistance in Italy assessed on *Helicobacter pylori* DNA sequences by TaqMan real-time polymerase chain reaction. *Aliment. Pharmacol. Ther.* 23 429–435. 16423002 10.1111/j.1365-2036.2006.02769.x

[B8] Garcia M. Raymond J. Garnier M. Cremniter J. Burucoa C. (2012). Distribution of spontaneous gyrA mutations in 97 fluoroquinolone-resistant *Helicobacter pylori* isolates collected in France. *Antimicrob. Agents Chemother.* 56 550–551. 10.1128/AAC.05243-11 22064536 PMC3256052

[B9] Hanafi A. Lee W. C. Loke M. F. The X. Shaari A. Dinarvand M. (2016). Molecular and proteomic analysis of levofloxacin and metronidazole resistant *Helicobacter pylori*. *Front. Microbiol.* 15:2015. 10.3389/fmicb.2016.02015 28018334 PMC5157799

[B10] Hunt R. H. Xiao S. D. Megraud F. Leon-Barua R. Bazzoli F. van der Merve S. (2011). *Helicobacter pylori* in developing countries. world gastroenterology organisation global guideline. *J. Gastrointestin. Liver Dis.* 20 299–304. 21961099

[B11] Ierardi E. Giorgio F. Losurdo G. Di Leo A. Principi M. (2013). How antibiotic resistances could change *Helicobacter pylori* treatment: a matter of geography? *World J. Gastroenterol.* 19 8168–8180. 10.3748/wjg.v19.i45.8168 24363506 PMC3857438

[B12] Iwańczak B. Borys-Iwanicka A. Biernat M. Gościniak G. (2016). Assessment of sequential and standard triple therapy in treatment of *Helicobacter pylori* infection in children dependent on bacteria sensitivity to antibiotics. *Adv. Clin. Exp. Med.* 25 701–708. 10.17219/acem/38554 27629844

[B13] Iwańczak B. Laszewicz W. Iwanczak F. Dzierzanowska-Fangrat K. Rozynek M. Dzierzanowska D. (2014). Genotypic and clinical differences of seropositive *Helicobacter pylori* children and adults in the Polish population. *J. Physiol. Pharmacol.* 65 801–807. 25554984

[B14] Karczewska E. Klesiewicz K. Wojtas-Bonior I. Skiba I. Sito E. Czajecki K. (2014). Levofloxacin resistance of *Helicobacter pylori* strains isolated from patients in Southern Poland, between 2006-2012. *Acta Pol. Pharm.* 71 477–483. 25265828

[B15] Klesiewicz K. Nowak P. Karczewska E. Skiba I. Wojtas-Bonior I. Sito E. (2014). PCR-RFLP detection of point mutations A2143G and A2142G in 23S rRNA gene conferring resistance to clarithromycin in *Helicobacter pylori* strains. *Acta Biochim. Pol.* 61 311–315. 24927236

[B16] Koletzko S. Jones N. L. Goodman K. J. Gold B. Rowland M. Cadranel S. (2011). H. pylori working groups of ESPGHAN and NASPGHAN evidence-based guidelines from ESPGHAN and NASPGHAN for *Helicobacter pylori* infection in children. *J. Pediatr. Gastroenterol. Nutr.* 53 230–243. 10.1097/MPG.0b013e3182227e90 21558964

[B17] Malfertheiner P. Megraud F. O’Morain C. A. Atherton J. Axon A. T. Bazzoli F. (2012). Management of *Helicobacter pylori* infection – the Maastricht IV/ Florence consensus report. *Gut* 61 646–664. 10.1136/gutjnl-2012-302084 22491499

[B18] Mégraud F. (2004). H. pylori antibiotic resistance: prevalence, importance, and advances in testing. *Gut* 53 1374–1384. 10.1136/gut.2003.022111 15306603 PMC1774187

[B19] Mégraud F. (2017). Time to change approaches to *Helicobacter pylori* management. *Lancet Gastroenterol. Hepatol.* 2 692–693. 10.1016/S2468-1253(17)30245-5 28781120

[B20] Miyachi H. Miki I. Aoyama N. Shirasaka D. Matsumoto Y. Toyoda M. (2006). Primary levofloxacin resistance and gyrA/B mutations among *Helicobacter pylori* in Japan. *Helicobacter* 11 243–249. 10.1111/j.1523-5378.2006.00415.x 16882327

[B21] Moore R. A. Beckthold B. Wong S. Kureishi A. Byan L. E. (1995). Nucleotide sequence of the gyrA gene and characterization of ciprofloxacin-resistant mutants of *Helicobacter pylori*. *Antimicrob. Agents Chemother.* 39 107–111. 10.1128/AAC.39.1.107 7695290 PMC162494

[B22] Odenbreit S. Swoboda K. Barwig I. Ruhl S. Boren T. Koletzko S. (2009). Outer membrane protein expression profile in *Helicobacter pylori* clinical isolates. *Infect. Immun.* 77 3782–3790. 10.1128/IAI.00364-09 19546190 PMC2738020

[B23] Phan T. N. Santona A. Tran V. H. Tran T. N. Le V. A. Cappuccinelli P. (2015a). High rate of levofloxacin resistance in a background of clarithromycin- and metronidazole-resistant *Helicobacter pylori* in Vietnam. *Int. J. Antimicrob. Agents* 45 244–248. 10.1016/j.ijantimicag.2014.10.019 25499186

[B24] Phan T. N. Tran V. H. Tran T. N. Le V. A. Santona A. Rubino S. (2015b). Antimicrobial resistance in *Helicobacter pylori*: current situation and management strategy in Vietnam. *J. Infect. Dev. Ctries.* 9 609–613. 10.3855/jidc.6942 26142670

[B25] Raymond J. Burucoa C. Pietrini O. Bergeret M. Decoster A. Wann A. (2007). Clarithromycin resistance in *Helicobacter pylori* strains isolated from French children: prevalence of the different mutations and coexistence of clones harboring two different mutations in the same biopsy. *Helicobacter* 12 157–163. 10.1111/j.1523-5378.2007.00486.x 17309753

[B26] Rimbara E. Noguchi N. Kawai T. Sasatsu M. (2012). Fluoroquinolone resistance in *Helicobacter pylori*: role of mutations at position 87 and 91 of GyrA on the level of resistance and identification of a resistance conferring mutation in GyrB. *Helicobacter* 17 36–42. 10.1111/j.1523-5378.2011.00912 22221614

[B27] Seck A. Burucoa C. Dia D. Mbengue M. Onambele M. Raymond J. (2013). Primary antibiotic resistance and associated mechanisms in *Helicobacter pylori* isolates from Senegalese patients. *Ann. Clin. Microbiol. Antimicrob.* 12:3. 10.1186/1476-0711-12-3 23298145 PMC3552979

[B28] Tacconelli E. Carrara E. Savoldi A. Harbarth S. Mendelson M. Monnet D. (2018). Discovery, research, and development of new antibiotics: the WHO priority list of antibiotic-resistant bacteria and tuberculosis. *Lancet Infect. Dis.* 18 318–327. 10.1016/S1473-3099(17)30753-3 29276051

[B29] Tankovic J. Petit J. C. Soussy C. J. (2003). Single and double mutations in gyrA but not in gyrB are associated with low- and high-level fluoroquinolone resistance in *Helicobacter pylori*. *Antimicrob. Agents Chemother.* 47 3942–3944. 10.1128/AAC.47.12.3942-3944.2003 14638505 PMC296230

[B30] Taylor D. E. Ge Z. Purych D. Lo T. Hiratsuka K. (1997). Cloning and sequence analysis of two copies of a 23S rRNA gene from *Helicobacter pylori* and association of clarithromycin resistance with 23S rRNA mutations. *Antimicrob. Agents Chemother.* 41 2621–2628. 10.1128/AAC.41.12.2621 9420030 PMC164180

[B31] Ubhayawardana N. L. Weerasekera M. M. Weerasekera D. Samarasinghe K. Gunasekera C. P. Fernando N. (2015). Detection of clarithromycin-resistant *Helicobacter pylori* strains in a dyspeptic patient population in Sri Lanka by polymerase chain reaction-restriction fragment length polymorphism. *Indian J. Med. Microbiol.* 33 374–377. 10.4103/0255-0857.158557 26068338

[B32] Vaziri F. Najar Peerayeh S. Alebouyeh M. Molaei M. Maghsoudi N. Zali M. R. (2013). Determination of *Helicobacter pylori* CagA EPIYA types in Iranian isolates with different gastroduodenal disorders. *Infect. Genet. Evol.* 17 101–105. 10.1016/j.meegid.2013.03.048 23567822

[B33] Versalovic J. Osato M. S. Spakovsky K. Dore M. P. Reddy R. Stone G. G. (1997). Point mutations in the 23S rRNA gene of *Helicobacter pylori* associated with different levels of clarithromycin resistance. *J. Antimicrob. Chemother.* 40 283–286. 10.1093/jac/40.2.283 9301997

[B34] Wang L. H. Cheng H. Hu F. L. Li J. (2012). Distribution of gyrA mutations in fluoroquinolone-resistant *Helicobacter pylori* strains. *World J. Gastroenterol.* 16 2272–2277. 10.3748/wjg.v16.i18.2272 20458765 PMC2868221

[B35] Wu W. Yang Y. Sun G. (2012). Recent Insights into Antibiotic Resistance in *Helicobacter pylori* Eradication. *Gastroenterol. Res. Pract.* 2012:723183. 10.1155/2012/723183 22829809 PMC3398622

