# Supplementary material for: Molecular Patterns of Resistance Among Helicobacter pylori Strains in South-Western Poland
Source: Front Microbiol. 2018 Dec 18;9:3154. doi: 10.3389/fmicb.2018.03154 (PMC6305312; doi:10.3389/fmicb.2018.03154)

Fluorograms

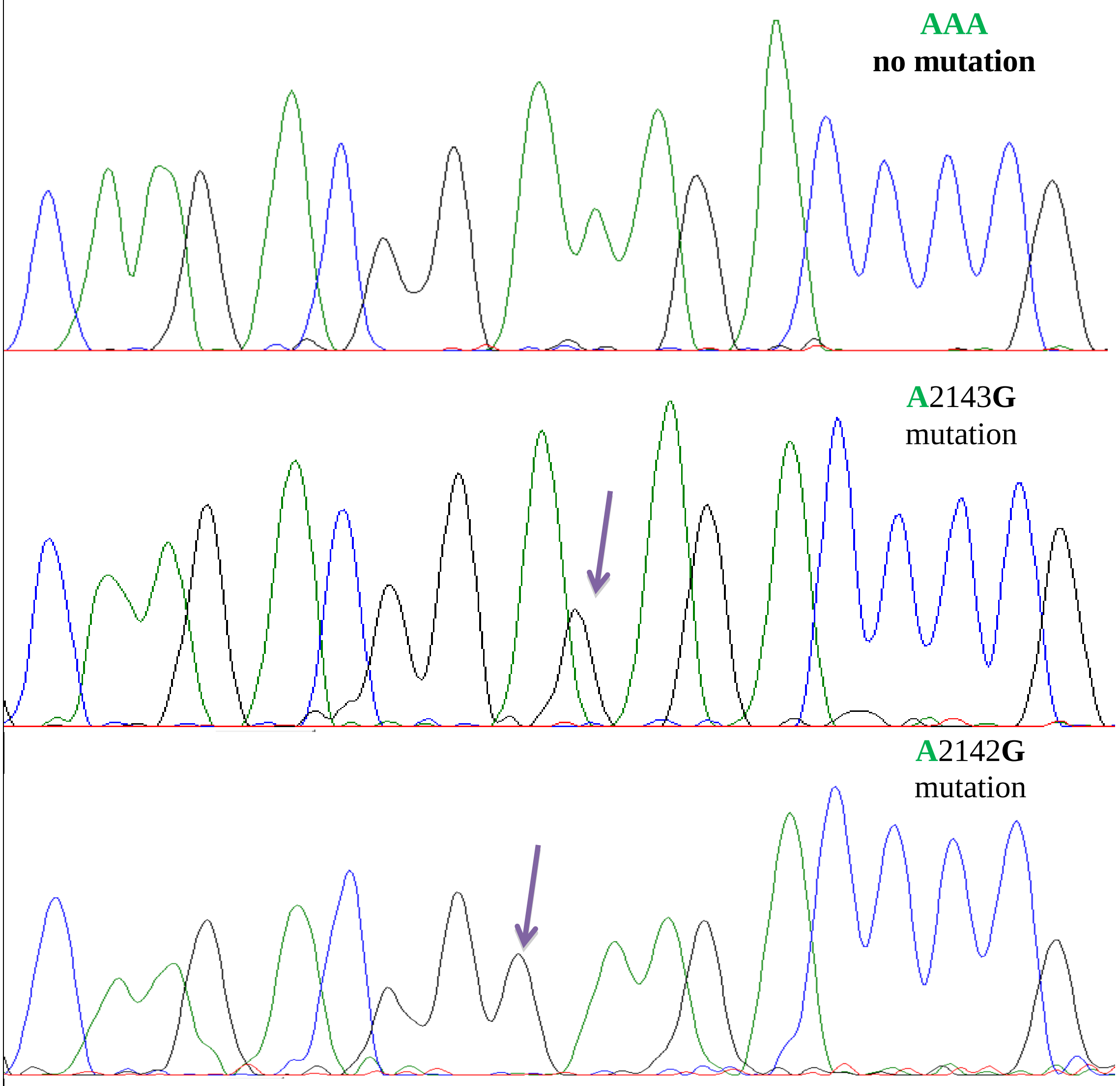

Fluorogram of gen's fragment 23s rRNA without mutations and with mutation in positions 2142 and 2143.

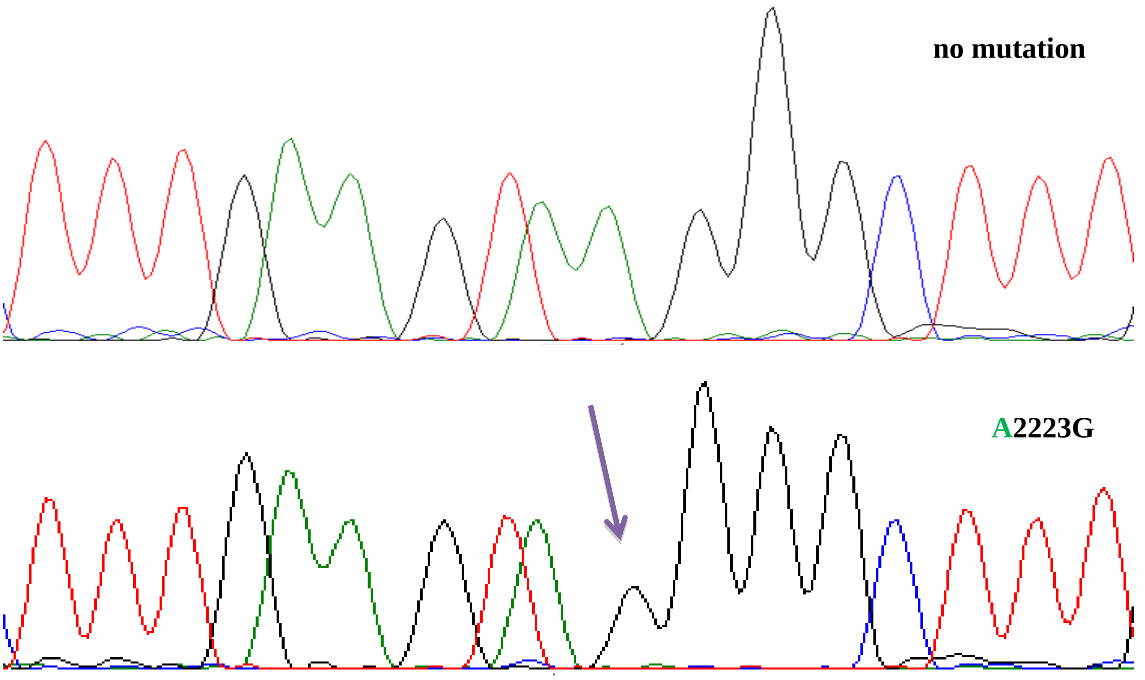

Fluorogram of gen's fragment 23s rRNA without mutations and with mutation in position 2223.

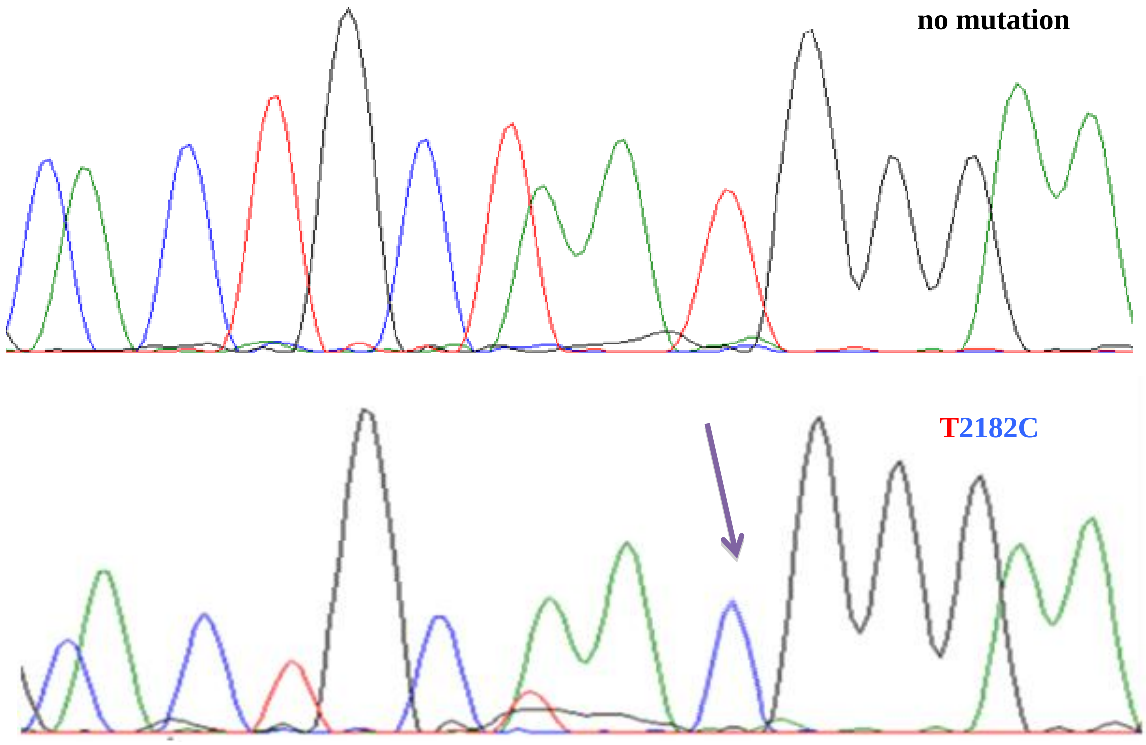

Fluorogram of gen's fragment 23s rRNA without mutations and with mutation in position 2182.

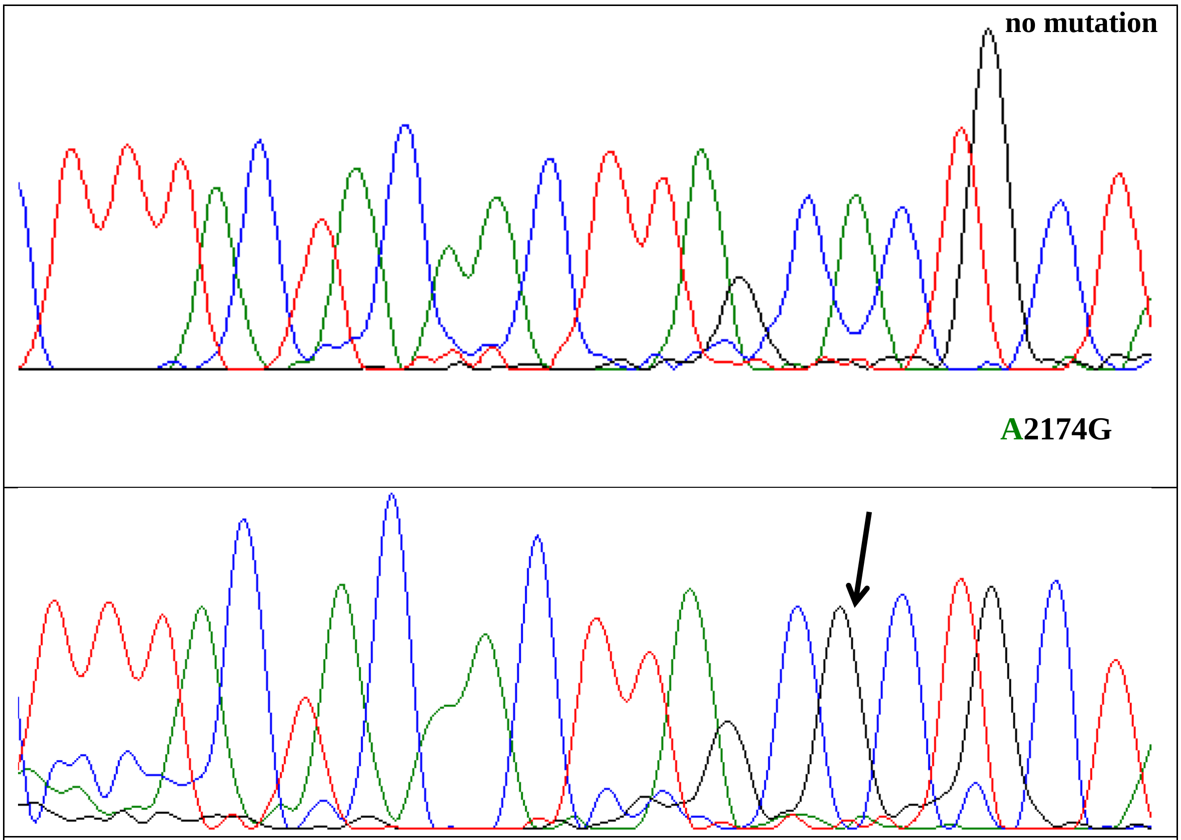

Fluorogram of gen's fragment 23s rRNA without mutations and with mutation in position 2174.

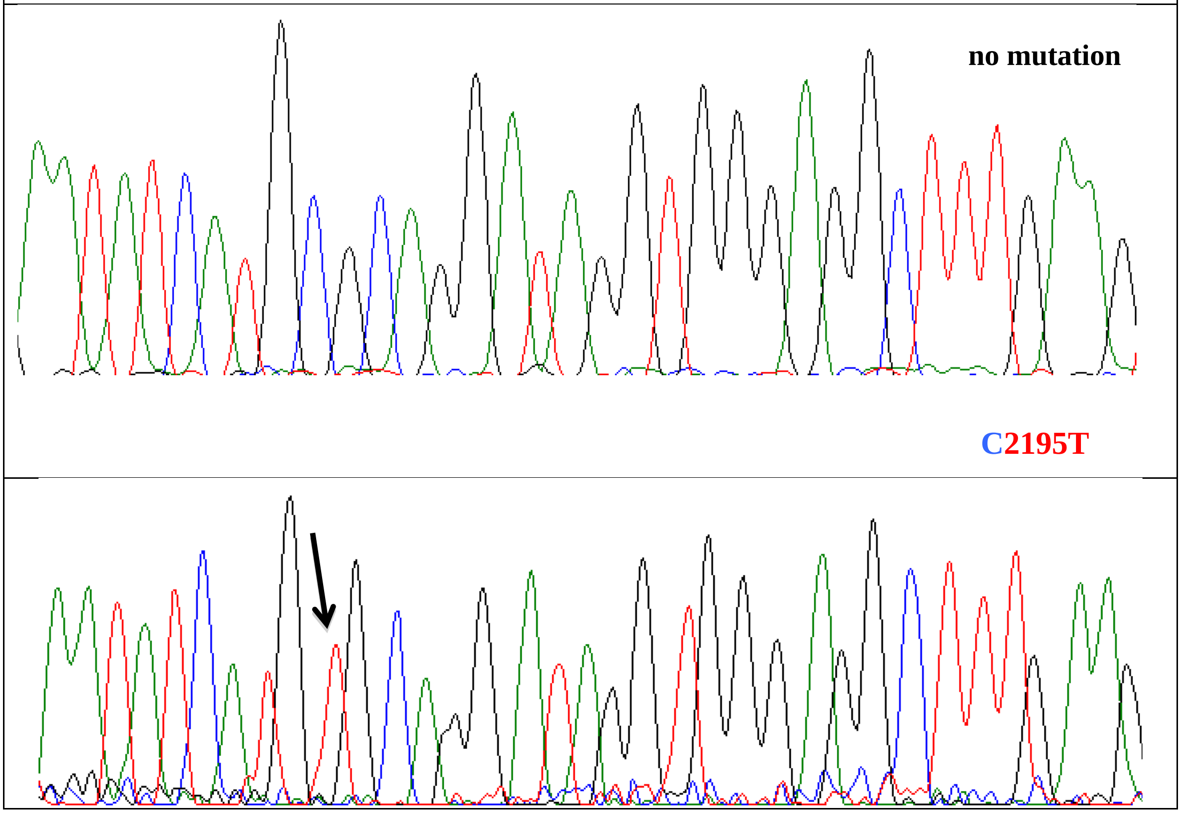

Supplement: Supplementary file 1 [file Data_Sheet_1.pdf]
